# Lithological and stress anisotropy control large-scale seismic velocity variations in tight carbonates

**DOI:** 10.1038/s41598-021-89019-4

**Published:** 2021-05-04

**Authors:** F. Trippetta, M. R. Barchi, E. Tinti, G. Volpe, G. Rosset, N. De Paola

**Affiliations:** 1 Dipartimento di Scienze Della Terra (DST), Sapienza Università di Roma, 00185 Rome, Italy; 2 Dipartimento di Fisica e Geologia, Università di Perugia, 06123 Perugia, Italy; 3 Istituto Nazionale di Geofisica e Vulcanologia, 00143 Rome, Italy; 4 Dipartimento di Matematica e Geoscienze, Università di Trieste, 34128 Trieste, Italy; 5 Department of Earth Sciences, Durham University, Durham, DH13LE UK

**Keywords:** Structural geology, Geophysics

## Abstract

Our knowledge of subsurface structures often derives from seismic velocities that are measured during seismic acquisition surveys. These velocities can greatly change due to lithological, fracture frequencies and/or effective pressure/temperature variations. However, the influence of such intrinsic lithological properties and environmental conditions at the large scale is poorly understood due to the lack of comprehensive datasets. Here, we analyze 43 borehole-derived velocity datasets of 3 end-member tight carbonate sequences from Central Italy, including massive pure limestone (Calcare Massiccio, CM), thick-layered (20–50 cm) pure limestone (Maiolica, MA), and thin-layered (2–20 cm) marly limestone (Calcareous Scaglia, CS). Our results show that the main rock parameters and environmental conditions driving large scale velocity variations are bedding and paleostresses, while mineralogical composition and current tectonic stress also play a role. For each of the 3 end-members, measured V_P_ values vary differently with depth, as the thin-layered CS units show a clear increase in Vp, while velocity slightly increases and remains constant for the thick-layered MA and massive CM units, respectively. Such observations show that velocities are affected by specific characteristics of lithological discontinuities, such as the thickness of bedding. Counterintuitively, larger Vp values were recorded in the deformed mountain range than in the undeformed foreland suggesting that higher paleo-stresses increase velocity values by enhancing diagenesis and healing of discontinuities. Our results thus demonstrate that large scale velocity variations are strictly related to variation of lithological properties and to the geological and tectonic history of an area. We suggest that such lithological and environmental controls should be taken into account when developing velocity and mechanical models for tectonically active regions of the Mediterranean Area, where earthquakes mostly nucleate and propagate through carbonate formations, and for resource exploration in fractured carbonate reservoirs.

## Introduction

Seismic properties of rocks are related to many parameters such as mineralogical composition, porosity, fabric anisotropy, density, pore type and shape that are usually investigated at laboratory-scale^1–14^. Moreover, crustal scale conditions such as stress and temperature^15–18^, fluid pressure^19,20^ or lithology lateral variations^21^, can also change resulting in complex seismic properties-depth relations according to lithology, rate of compaction, grain types and amount of cementation^22,23^. These variations are particularly relevant to carbonate rocks that play a strategic role for resource exploration accounting for more than 60% of the world's proven hydrocarbon reserves^24^. Accurate estimates of seismic velocity of carbonates are critical for a correct interpretation and depth conversion of seismic profiles, which drives oil and gas exploration. Moreover, in the tectonically active regions of the Mediterranean Area, earthquakes mostly nucleate and propagate through carbonate formations^25–30^. Detailed crustal scale seismic velocity profiles are needed for the localization of earthquakes, which is necessary for seismic hazard modeling at local and regional scale.

In the Apennines, marine carbonate successions were deposited on the southern continental passive margin of the western Tethys Ocean, during the Mesozoic-Early Tertiary. Hence, they show a range of facies, from platform (shallow water) to basin (deep water) units, and varying intensity of fracturing from highly deformed to relatively undeformed areas. Extensive exposure at the surface and many drilled exploration wells make this region one of best studied case-story for carbonate rocks^1,31–34^ worldwide. In particular, the availability of a large volume of well data^35^ allows the analysis of seismic velocity variations and its dependency on key parameters over a relatively large area.

**Figure 1:**
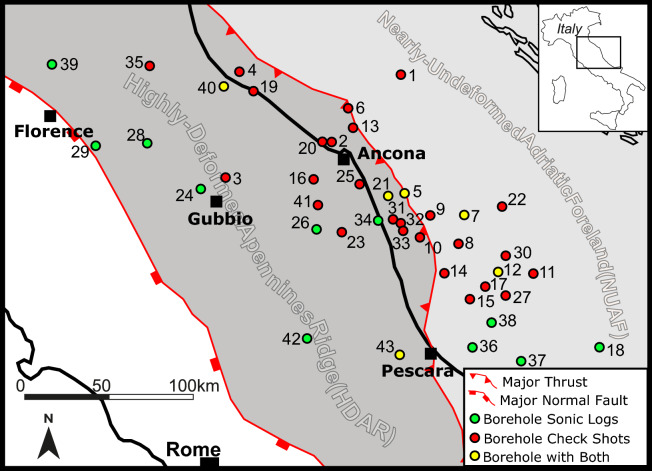
Schematic structural map where Highly Deformed Apennine Ridge (HDAR) and Nearly-Undeformed Adriatic Foreland (NUAF) are shown with the location map of the boreholes analyzed in this work. Green, Red and Yellow dots are for boreholes where, respectively, sonic logs, check shots or both were available. Numbers indicate the wells: 1 Alessandra1, 2 Barbarossa1, 3 Burano1, 4 Canopo1, 5 Conrad1, 6 Cornelia1, 7 Daniel1, 8 Dante1, 9 Donald1, 10 Dora1, 11 Edgar1, 12 Edmond1tris, 13 Elga1, 14 Emilio3, 15 Emma1, 16 Esino1, 17 Esmeralda1, 18 Famoso1, 19 Gabicce1, 20 GabriellaMare1, 21 Manila1, 22 Mizar1, 23 Mogliano1, 24 Monte Civitello1, 25 Musone1, 26 Paterno 1, 27 Patrizia1, 28 PieveSantoStefano1, 29 Pratomagno1, 30 Rigel1bis, 31 SanGiorgio1, 32 SanGiorgio2, 33 SanGiorgio5, 34 SantaMariaTerra3, 35 Sarsina1, 36 Silvana 1 , 37 Silvia 1 , 38 Spinello Mare1, 39 Suviana1, 40 Tavullia1, 41 Treia1, 42 Varoni1, 43 Villadegna1. Map was generated by using Inkscape (1.0.2, https://www.inkscape.org) graphic editor. Major fault traces are those reported by^81^, while wells have been positioned by using Matlab (R2020b, https://www.mathworks.com) following Latitude and Longitude information^35^ for each well.

In this paper, we compare and analyze carbonate-velocity data from 43 boreholes drilled in Central Italy, spanning from the highly deformed Apennine ridge sector to the relatively undeformed Adriatic foreland (Fig. 1). For each borehole, we analysed three thicker, laterally homogeneous and continuous litho-stratigraphic units of the Umbria-Marche multilayer, representative of tight carbonates: massive pure limestone (Calcare Massiccio, CM), thick-layered pure limestone (Maiolica, MA), and thin-layered marly limestone (Calcareous Scaglia, CS). We then compared the seismic-velocities derived from each borehole to obtain the mean velocity variations for the different carbonate facies and tectonic areas^31,32^.

## Study area

This paper is focused on the study of the seismic properties of marine tight carbonate rocks of the Mesozoic-Early Tertiary Umbria-Marche succession^36^. The carbonate-dominated multilayer is an arc-shaped eastward-verging fold-and thrust belt, (Fig. 1) representing the backbone of the Outer Northern Apennines^37,38^. Within the sedimentary cover of the Umbria-Marche Apennines, the carbonate multilayer overlies a thick (ca. 1500 m) sequence of Late Triassic evaporites (Burano Fm.,^39^) and is covered by eastward youngling successions of syn-orogenic turbidites^32,40^.

Since more than one century, the stratigraphic continuity and the quality of outcrops of the Umbria-Marche carbonate succession have attracted a wide community of international scientists, promoting many paradigmatic stratigraphic case-studies (e.g. magnetic stratigraphy, sequential stratigraphy, see^41^ for a review).

In the last decades of the XX century, the Umbria-Marche Apennines were also object of exploration by oil industry, promoting the acquisition of an irregular network of seismic reflection profiles and the drilling of deep boreholes^31,42,43^, which provided new data on the subsurface setting of the region. These wells drilled the carbonate succession in different structural position, such as along the mountain ridge, in its foothills and in the nearly-undeformed foreland. In the foreland, the pre-orogenic carbonate multilayer is covered by thick (up to 10 km) Neogene-Quaternary syn-tectonic deposits, mainly consisting of siliciclastic turbidites^43^. In this work, we extracted and elaborated data collected in 43 vertical wells (see Fig. 1 and Supplementary Table S1), 27 of which were drilled in the western part of the region (Highly-Deformed Apennines Ridge, HDAR in Fig. 1), where the carbonate multilayer is involved in the compressional structures of the Apennines. The other 16 wells are located in the eastern foreland region (Nearly-Undeformed Adriatic Foreland, NUAF in Fig. 1), where the carbonates have experienced little horizontal shortening^44^. No significant fluid overpressures are reported for the analyzed boreholes.

**Figure 2:**
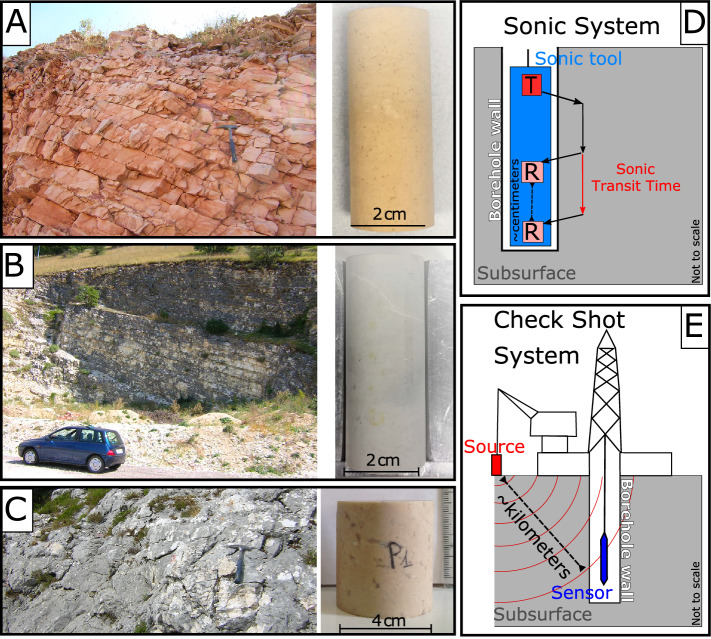
Mesoscale (left side) and small-scale samples (right side) of the analyzed lithologies. (**A**) Massive limestone—Calcare Massiccio (CM), (**B**) Layered limestone—Maiolica (MA) and (**C**) Layered marly limestone—Calcareous Scaglia (CS). (**D**) Sonic System sketch; note that the distance between the transmitter (T) and the two receivers (R) is in the order of centimeters. (**E**) Check shot system sketch; note that the distance between the source and the sensor is in the order of kilometers.

The Umbria-Marche carbonate succession consists of many different formations, with strong vertical variability, recording carbonate facies variations during a 150 Myrs-long geologic history^45^. For our study, we decided to select three representative major lithological units, from bottom to top, the Calcare Massiccio Formation (Fm.), the Maiolica Fm. and the Calcareous Scaglia (Fig. 2). The Calcare Massiccio Fm. (Early Jurassic) is made by massive limestones, deposited in a shallow water, carbonate platform environment. The average drilled thickness is ~ 700 m and the maximum drilled thickness of ~ 1400 m (Table S2). The Maiolica Fm. (Late Jurassic-Early Cretaceous) is made by thick-layered (20–50 cm) “pure” limestone (less than 5% of clay and cherts), deposited in a pelagic marine environment with a average drilled thickness of 200 m and a maximum drilled thickness of ~ 600 m (Table S2). The Calcareous Scaglia (Late Cretaceous-Early Tertiary) includes the two adjacent Formations: the Scaglia Bianca and the Scaglia Rossa Fms., with similar lithology but different color. The Calcareous Scaglia is made by thin-layered (5–20 cm) “marly” limestone (generally less than 20% of clay and cherts), deposited in a pelagic marine environment, with an average drilled thickness of ~ 400 m and a maximum drilled thickness of ~ 600 m (Table S2). All the three units are characterized by low porosity^43^ (generally lower than 5%). Hereafter, the three lithological units considered in this study are simply referred to as: *Calcare Massiccio* (CM), *Maiolica* (MA) and *Calcareous Scaglia* (CS).

Among the above described lithological units, the CS is by far the less homogeneous, showing relevant vertical variability, due to interlayered marly layers (up to 20 cm thickness) and resedimented calcarenites intervals (up to 20 m thickness)^46^. Although it is not easy to recognize such lithological variations in the CS by the well data used in this study, it is generally observed that the clay content is higher in the upper Tertiary portion than in the lower Cretaceous part.

## Results

In this section, we report the seismic velocities of the three considered litho-stratigraphic units (CM, MA and CS), acquired with two techniques: sonic logs and check shots.

The sonic log technique measures the difference in the arrival times (Dt) of the acoustic wave at two receivers, usually positioned at a distance of 3 and 5 ft, with respect to the source^47^ (Fig. 2D). The average frequency is between 20 and 40 kHz^47–50^ and the instrumental error is below 0.4 μs/ft^47^. Dt is generally expressed in μs/ft, which represent the elapsed time for every foot, and the conversion Vp [km/s] = 304.8/Dt [μs/ft] is used to obtain the velocity values in km/s. Hereafter, we refer to the velocity derived from the sonic log arrival time with the terms “Sonic Log Velocity” (SLV).

The check shot time is the transit time measured between the surface and a downhole receiver^47–50^ (Fig. 2E). Differences of arrival time at different depths define the velocity of the interval. Thus, the velocities acquired using this technique cover kilometers of rocks and the average frequency is in the range of 1 to 10 Hz. The instrumental error depends on the depth and on the absolute velocity values and in the literature is considered to be 1–2% of the Vp values^47^. For simplicity, hereafter we refer to the velocity derived from the check shot time with the terms “Interval velocity” (IV).

**Figure 3:**
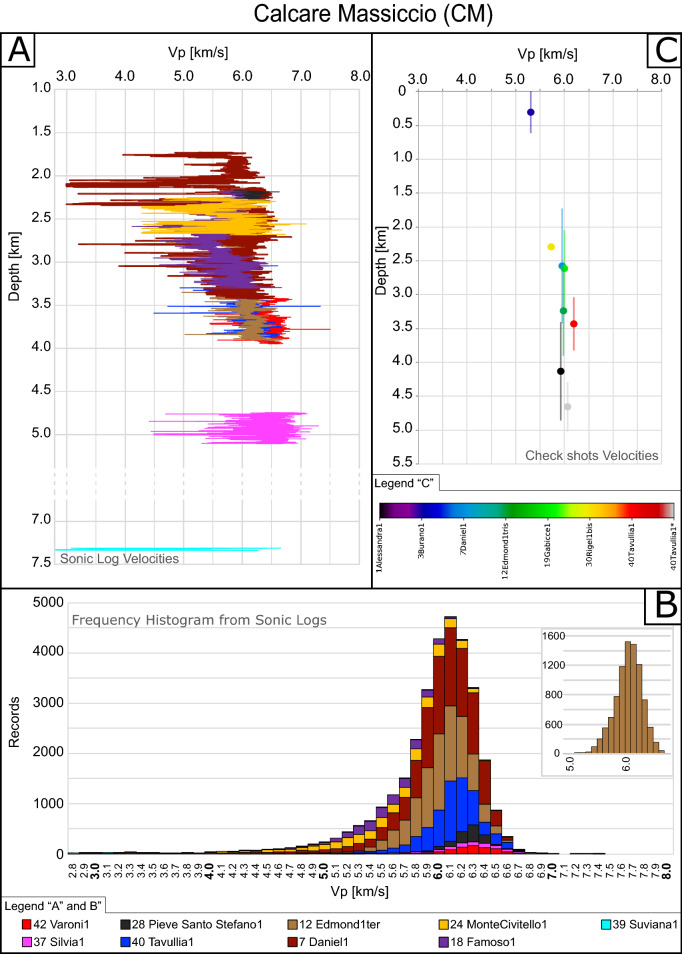
(**A**) In situ Vp sonic logs (SLV) depth profiles of boreholes through Calcare Massiccio (CM). (**B**) Velocity frequency cumulative histograms of the boreholes in panel A; y-Axis shows the number of times (frequency) that a certain velocity was recorded. *Tavullia 1 drilled the CM twice: from 3044 to 3.830 m and from 4298 to 5025 m. Inset shows the frequency analysis of a representative single well (Edmond1tris). (**C**) Interval velocities (IV) depth profile for available boreholes for the CM portions where vertical bars indicate the drilled thickness. Note that IV instrumental error is in the order of 2% (based on estimates in previous literature)^47^ and that error bars are reported in Fig. 6.

**Figure 4:**
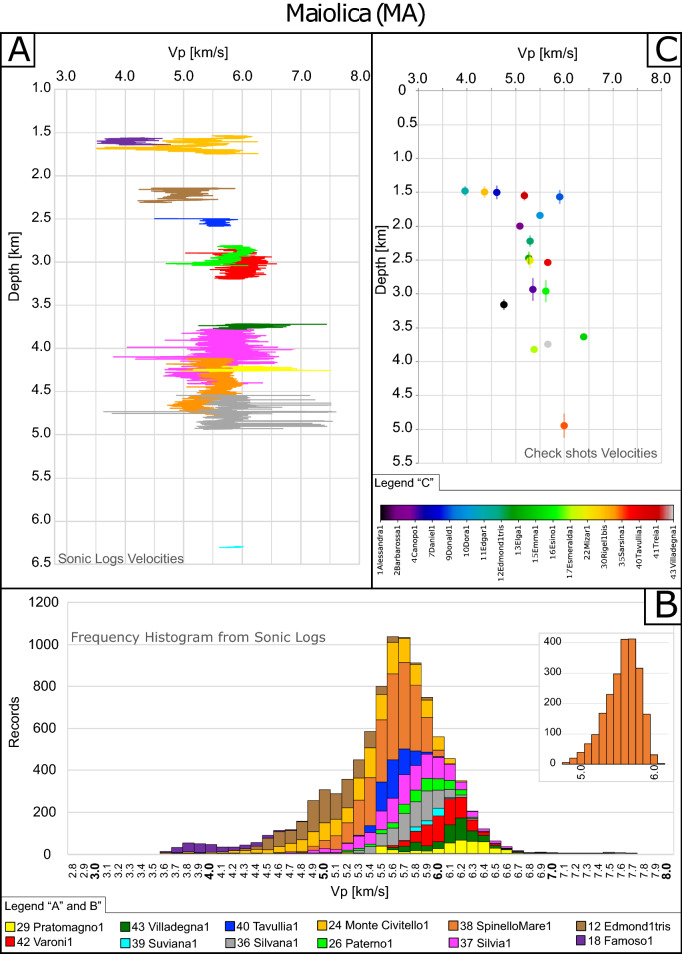
(**A**) In situ Vp sonic logs (SLV) depth profiles of boreholes through Maiolica (MA). (**B**) Velocity frequency cumulative histograms of the boreholes in panel A; y-Axis shows the number of times (frequency) that a certain velocity was recorded. Inset shows the frequency analysis of a representative single well (SpinelloMare1). (**C**) Interval velocities (IV) depth profile for available boreholes for the MA portions where vertical bars indicate the drilled thickness. Note that IV instrumental error is in the order of 2% (based on estimates in previous literature)^47^ and that error bars are reported in Fig. 6.

**Figure 5:**
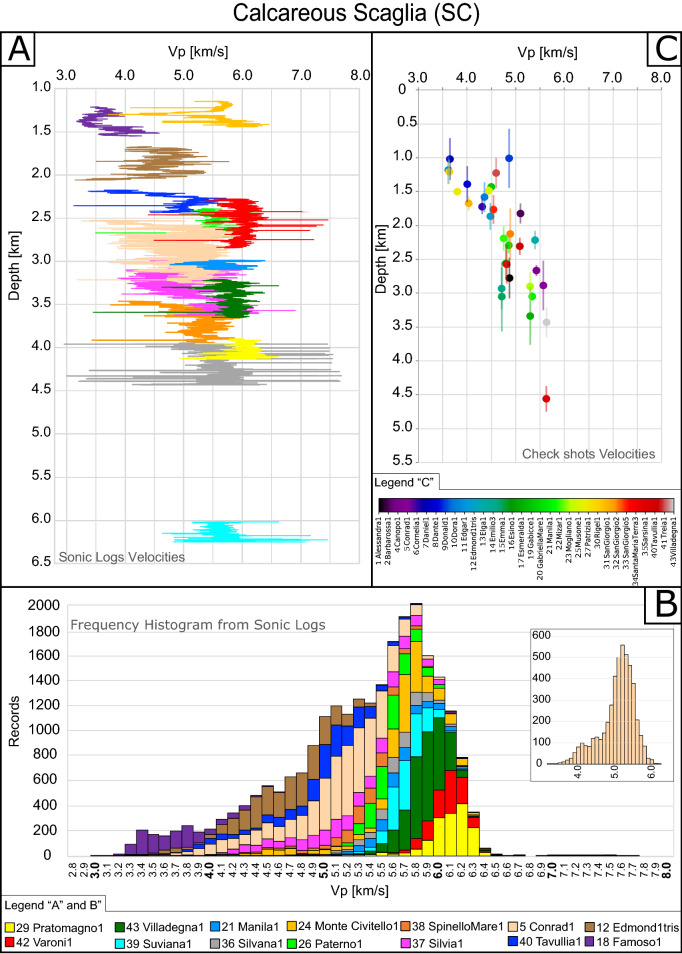
(**A**) In situ Vp sonic logs (SLV) depth profiles of boreholes through Calcareous Scaglia (CS). (**B**) Velocity frequency cumulative histograms of the boreholes in panel (**A**); y-Axis shows the number of times (frequency) that a certain velocity was recorded. To be noted that some SLV reports distinguished the upper Tertiary section from the lower Cretaceous one: for these wells, the Cretaceous CS is always faster than Tertiary CS, which is usually characterized by a greater marly fraction (e.g.^77^). For simplicity, for the boreholes where Tertiary and Cretaceous portions of CS were distinguished, we only reported the weighted mean velocity of the whole interval. Inset shows the frequency analysis of a representative single well (Conrad1). (**C**) Interval velocities (IV) depth profile for available boreholes for the CS portions where vertical bars indicate the drilled thickness. Note that IV instrumental error is in the order of 2% (based on estimates in previous literature)^47^ and that error bars are reported in Fig. 6.

For all the boreholes and for each litho-stratigraphic unit we will illustrate:

- the SLV vs depth (Figs. 3A, 4A and 5A).
- the velocity distribution expressed in terms of the number of times (frequency) that a certain velocity was registered from the SLV (from 3 to 6 measurements each meter) along the available boreholes. The histograms derived from this frequency analysis give a more complete image of the statistical distribution of the velocity values (Figs. 3B, 4B and 5B) with respect to simply averaging the Vp values. To note here that the analysis of frequency of appearance can be slightly biased by the number of wells drilled at each depth.
- the IV vs. depth (Figs. 3C, 4C and 5C).

### Calcare Massiccio (CM)

For the CM unit, we analyzed SLV data derived from 9 wells and IV data from 7 wells (Supplementary Table S1 and Fig. 3).

None of the analyzed sonic logs shows any significant increase with increasing depth within the single well (Fig. 3A), in contrast with normal trends of most of the lithologies showing constantly increasing velocities with depth^11,23,51,52^.

The frequency analysis for CM is reported in Fig. 3B. The velocity distribution of each single well is generally unimodal (example in Fig. 3B inset). The clear unimodal velocity distribution of the SLV in the CM defines a very homogeneous lithology, from the seismic properties point of view. On the contrary, increasing velocities with depth within a single well would result in a heterogeneous frequency distribution of velocities without any clear modal value.

By looking at the velocity distribution of all the wells (Fig. 3B), the recorded velocities within 1std deviations are comprised between 5.4 and 6.4 km/s, with a cumulative unimodal distribution. The modal value is 6.1 km/s being in good agreement with the mean value of the distribution (5.9 km/s). Varoni 1, Silvia 1 and Pieve Santo Stefano 1 wells show the highest modal values (~ 6.3 km/s) and dolomitization of CM is always documented in the composite log of these wells^35^. The Suviana 1 well that drilled CM at the deepest depth of the dataset shows the smaller mean velocity (4.1 km/s). However, this is a quite anomalous value since it is much lower with respect to all the other boreholes and thus probably related to some problems during the acquisition that affected SLV measurements^48,50^. This is confirmed by the total loss of fluids reported for the depth interval 7313–7700 in the composite sonic log^35^.

The pattern of IV acquired for CM also shows a very slight depth dependence (Fig. 3C) mainly related to the two relatively low velocity data recorded in Burano 1 and Riegel 1 bis wells that drilled the CM at the shallowest depths of the entire dataset. Compared to SLV data, IV data available for CM (Fig. 3C), show a narrower velocity range, comprised between 5.3 and 6.2 km/s. However, this velocity range is in agreement with most of the SLV data (Fig. 3A,B). Moreover, IV data are also in good agreement with literature laboratory measurements on relatively small, unfractured samples of the same lithology, showing no pressure (i.e. depth) dependence and values of^2^ ~ 6.1 km/s. Laboratory measurements on dolostone samples generally recorded higher values, up to^53^ 7.3 km/s, in agreement with the higher SLV and IV recorded on the dolomitized portions of CM.

### Maiolica (MA)

For the MA unit, we analyzed SLV data derived from 12 wells and IV data from 18 wells (Supplementary Table S1 and Fig. 4).

Excluding the mean SLV value of the Monte Civitello 1, the analysis of the general trend regarding all the mean SLV values (Fig. 4A), shows a slight depth-dependence for wells that drilled MA at depth < 3 km, whilst mean SLV of deeper boreholes do not show any clear further increase in the recorded mean velocities by increasing depth (Fig. 4A).

The frequency analysis of single wells (example in Fig. 4B inset), is consistent with these observations, showing unimodal distributions with a quite clear modal velocity. The frequency histograms of all the recorded velocities (Fig. 4B) show that Vp within 1std deviations are in the range 5.0–6.0 km/s, with a quite unimodal distribution, where the most frequently recorded velocity is 5.7 km/s and the weighted mean velocity is 5.5 km/s. The frequency distribution (Fig. 4A) also shows a small tail toward the lower velocities (left side) where the Famoso 1 well shows recorded velocities in the range of 3.6–4.7 km/s with a modal value of 3.9 km/s. On the right side of the diagram, most of the highest velocities for MA have been measured in the Pratomagno 1, Villadegna 1 and Varoni 1wells (modal value ~ 6.2 km/s).

IV data range are in a quite larger interval, from ~ 4.0 to ~ 6.5 km/s (Fig. 4C), with respect to SLV data, while the pattern seems to be slightly dependent on depth (in particular for depths < 3 km) in agreement with SLV. IV data show a sparse distribution at shallow depth that is actually confirmed by the wide Vp range recorded by SLV on the two shallowest wells (Fig. 4A).

### Calcareous Scaglia (CS)

For the CS unit, we analyzed SLV data derived from 14 wells and IV data from 32 wells (Supplementary Table S1 and Fig. 5).

SLV within the single wells show a detectable increase in velocity with increasing depth. Many wells, particularly the shallower ones (Famoso 1, Edmond 1 ter, Tavullia 1 and Paterno 1), show a regular trend of increasing SLV with increasing depth (Fig. 5A).

The frequency analysis of the stacked SLV (Fig. 5B) reflects the distribution observed for most of the single wells (example in Fig. 5B inset). The frequency distribution for each sonic log is still consistent with a unimodal distribution (Fig. 5B inset), as observed for CM and MA, but the tail of data toward the lower velocities is more evident with respect to MA (Fig. 4B) in the cumulative histogram (Fig. 5B) where Vp within 1std deviations are in the range 4.6–5.9 km/s.

Consequently, for CS the modal value (5.8 km/s) is different with respect to the weighted mean (5.3 km/s). In analogy with what observed for MA, most of the lower velocities were recorded in the Famoso 1 well, whilst higher velocities have been measured in the Pratomagno 1, Villadegna 1 and Varoni 1wells (modal value ~ 6.2 km/s).

The IV measured in the 32 available wells (Fig. 5C) ranges from 3.6 to 5.8 km/s, with a rather clear depth dependence (Fig. 5C). As observed for MA, a quite wide Vp range is recorded at shallow depths. Velocity of CS increases with depth being this increase larger in the shallower portion and progressively lower at depth in agreement with literature data on most of the lithologies^11,23,54^.

## Discussion

A large number of papers in literature is focused on the factors that can influence the seismic velocities. For example, several authors investigated the role of pore pressure^53,55–59^, fabric anisotropy^52,53^ and fractures^5,60–63^ mainly starting from laboratory measurements conducted on centimeters-scale samples. Moreover, measurements of Vp values can be strongly influenced by the angle between the raypath and the bedding anisotropy, this effect being particularly important for IV measurements. Keeping in mind that all these factors have a large influence on seismic velocity in particular at local scale, in the following we are going to compare and discuss seismic velocity data coming from a large area. This analysis is aimed to investigate the crustal-scale changes in seismic velocities in two adjacent structural domains by meaning of a very large number of measurements recorded on the 43 analyzed boreholes.

### Influence of depth

**Figure 6:**
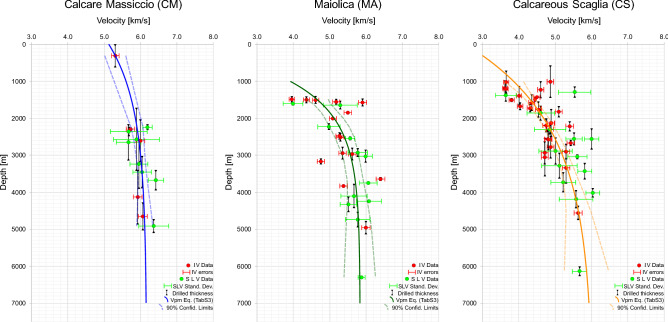
Mean velocities vs mean depths for boreholes that drilled Calcare Massiccio, Maiolica and Calcareous Scaglia. Green symbols are for Sonic logs data (SLV), Red symbols are for Check shot (IV). Vertical black bars indicate the thickness of each lithology drilled by the wells. Horizontal red and green bars represent respectively the IV data errors (2% following^47^) and the standard deviations of the SLV data. Colored (blue, green and orange) lines for each formation have been drawn following the equations reported in Supplementary Table S3 and the dashed lines represent the 90% confidence limit derived from such analysis. To infer the weighted best fit with an exponential function, the objective function implemented in Matlab has been applied to the available data. The procedure then gives the best coefficients (Supplementary Table S3) by minimizing the sum of square errors.

In order to compare results from SLV and IV methods, we calculated the mean velocity and the mean depth for each borehole for each of the three analyzed carbonate units, i.e. CM, MA and CS (Fig. 6). When both SLV and IV were available, we kept the SLV data due to the larger number of measurements for the same drilled interval with respect to IV. However, performed sensitivity tests showed that using IV data the following results would not significantly change since the two measurements show, mainly, similar results (see Supplementary material).

The SLV and IV values obtained are fairly consistent (Fig. 6), showing that a frequency-related bias^8,47^ was not present. The similar velocity values obtained by the two methods suggest that the different sampling frequencies (in the order of Hz for IV and kHz for SLV) do not significantly affect the results.

In particular, averaged SLV and IV both show the same dependence with depth, especially for the MA and CS units (Fig. 6); this observation allows us to merge the SLV and IV datasets.

For the MA and CS, the shallowest available data are at ~ 1500 m and at ~ 1000 m, respectively (Fig. 6), while for CM, only one borehole datum is available above 2000 m. To mitigate against the lack of borehole data in this depth range, we referred to laboratory measurements from the literature recorded on CM samples at increasing confining pressure^2^. Laboratory data^2^ show a Vp range of 5.8–6.2 km/s at confining pressures corresponding to depth < 2000 m (i.e. < 50 MPa, assuming a lithostatic load of 25 MPa/km). We didn’t use the laboratory data for the best-fit analysis, however, their good agreement with the borehole dataset, allow us to consider the shallow borehole data as reliable.

In agreement with literature and theory^8,23,47,54,64^, Vp increase with depth for all the three carbonate units, being well-fitted by the Athy’s-type exponential law^54^. The weighted best-fit curves are shown in Fig. 6 together with the 90% confidence limits, while equation parameters are reported in Supplementary Table S3.

It is well recognized that the Vp depth-dependency is controlled by the decrease in porosity^8,23,47,54,64^. The reduction of porosity that leads to the increase in Vp with depth is mainly due to compaction^23^ related to two main processes: chemical compaction by pressure solution and cementation, and mechanical compaction by pore space and fracture closure^65^.

Chemical compaction by pressure solution is enhanced by the presence of clays in carbonates. Accordingly, stylolites are rare in CM whilst are widespread in MA and particularly CS^66^, being the clay content much higher in CS than in MA and CM^67^.

The second chemical process is cementation that is mainly affected by temperature, which increases progressively with depth. Data in Fig. 6 show that the influence of depth is negligible for CM becoming also negligible for MA and CS below ~ 3.5 km, whilst temperatures surely keep increasing below this depth. We thus speculate that cementation should not be a factor affecting the observed variations of Vp in these formations.

Mechanical compaction is the main compaction mechanism acting in the upper portions of forming sedimentary basins^23^. It consists in the rearrangement of grains due to the response to loading and in the closure of suitably oriented planar anisotropy in the rocks, such as strata boundaries, fractures, or stylolites^54^. All the formations analyzed in this paper have similar low porosity^2,43^, but different mean layer thickness, being CM massive, MA thick-bedded (20–50 cm) and CS thin-bedded (5–20 cm). Fracture spacing is known to be directly proportional to the layer thickness^68,69^. Hence, assuming similar mechanical properties, thinner layers will develop a more pervasive fracture pattern than thicker layers. We interpret the observed weak depth-dependence of Vp values in the massive CM, as due to the presence of less mechanical discontinuities than in the MA and CS. On the contrary, mechanical discontinuities in the MA and CS formations, such as bedding surfaces, closely spaced fractures and stylolitic surfaces, are progressively closed with increasing depth due to the increase of the lithostatic load (i.e. confining pressure)^2,54,65^. Such reduction in structural porosity may explain the progressive increase of the recorded velocity^52^ in the MA and CS formations at shallow depth, while this effect is obviously absent in CM since few discontinuities are present (Fig. 6). This interpretation is consistent with the recorded low velocity values in the CS and MA units where, at shallow depth, a higher percentage of open fractures is likely to be present with respect to CM (see also Fig. 6). At larger depth, the increasing lithostatic load tends to close most of the fractures^2^ (if present), hence explaining the progressively smaller depth-dependence of velocity observed in all three formations (Fig. 6).

### Influence of tectonic deformation

Our dataset comprises velocity measurements from boreholes drilled in two different structural domains of the Umbria-Marche region (Fig. 1). The two domains are: the inner domain, including the Apennines fold-and-thrust belt, which experienced intense compressional and extensional deformation (Highly-Deformed Apennines Ridge, HDAR); and the outer foredeep/foreland domain, which only experienced the distal effects of the orogenic contraction (Nearly-Undeformed Adriatic Foreland, NUAF).

Past and presently active deformation change the petrophysical properties of rocks, and consequently their seismic velocities^2,70–73^. To investigate whether structural position plays a role in controlling the differences in velocity, we calculated the areal mean velocity values for CM, MA and CS units for the two structural domains (Supplementary Table S2).

MA and CS units show a sensible dependence on the structural position, whilst velocities of CM units are not much affected showing similar values of 6.0 km/s and 5.9 km/s for the HDAR and the NUAF, respectively. In particular, for MA the areal mean velocity is lower (5.4 km/s) in the NUAF and higher (5.8 km/s) in the HDAR, similarly to what observed for CS, with velocities of 4.7 km/s in the NUAF and 5.1 km/s in the HDAR. These areal mean data are in agreement with those derived from interval velocities reported in previous studies^18,31–33^, however, the difference in velocity between the two domains has never been discussed before. Mean depths have also been calculated for the two structural domains, being those of the HDAR larger with respect to NUAF for MA and CS (Supplementary Table S2).

**Figure 7:**
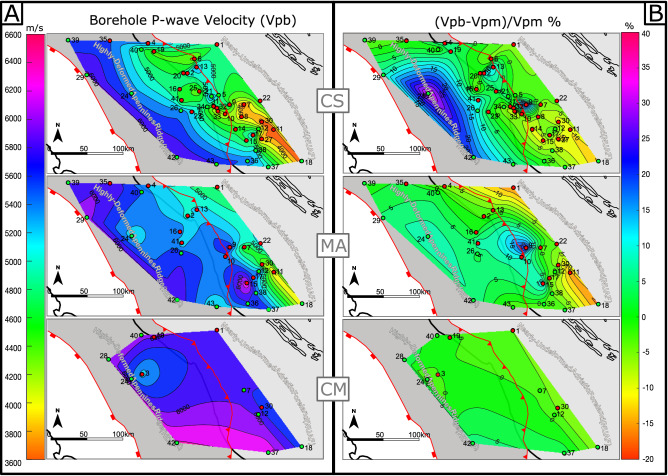
(**A**) Contour maps of the mean Vp recorded in the wells that drilled Calcare Massiccio (CM), Maiolica (MA) and Scaglia Group (SC). (**B**) Percentage Differences between the actual P-wave velocity (Vpb) recorded in each well and the modeled P-wave velocities (Vpm) using equations of Supplementary Table S3 for each investigate group (CM, MA and CS). Maps were generated by using Matlab (R2020b, https://www.mathworks.com) and then edited with Inkscape (1.0.2, https://www.inkscape.org) graphic editor.

In Fig. 7A, we report 2D contours of the velocities recorded in the analyzed wells for each formation. These maps show a marked decrease of the velocities from West (HDAR) to East (NUAF). Since carbonates are deeper in the HDAR, this decrease should be related to the decreasing lithostatic load moving toward NUAF. Consequently, before discussing results from the two structural domains, the data must be corrected to remove the effect of the present lithostatic load on the mean velocities (Fig. 6). Hence, we calculated a modeled velocity (Vpm) from equations reported in Supplementary Table S3 by using the mean depth of each well for each formation. We then calculated the differences (anomalies) between the measured (Vpb) and the modeled (Vpm) borehole velocity for each formation. We normalized this difference by Vpm, to obtain the percentage of variation in velocity mapped in Fig. 7B. These contour maps show negligible (always < 5%) variations for CM, while larger velocities are recorded for MA and CS in HDAR with respect to NUAF, being this particularly evident for CS. This trend can be related to the larger stress experienced in the mountain belt (HDAR) with respect to the foreland (NUAF)^18,74–76^.

Of particular relevance is the correspondence between the positive velocity anomalies and the main front of the Apennine chain. Such observations can be explained by the effect of the total stress field. In fact, both vertical lithostatic load and horizontal tectonic compression can promote fracture porosity in a rock volume that could decrease seismic velocities^46^ in particular at shallow depth. However, at the same time, this will enhance hydrothermal fluid circulation with pressure solution processes. Such processes can in turn significantly reduce rock porosity^52^ by promoting fracture healing and cementation^51^, which will increase the seismic conductivity of the involved lithologies.

**Figure 8:**
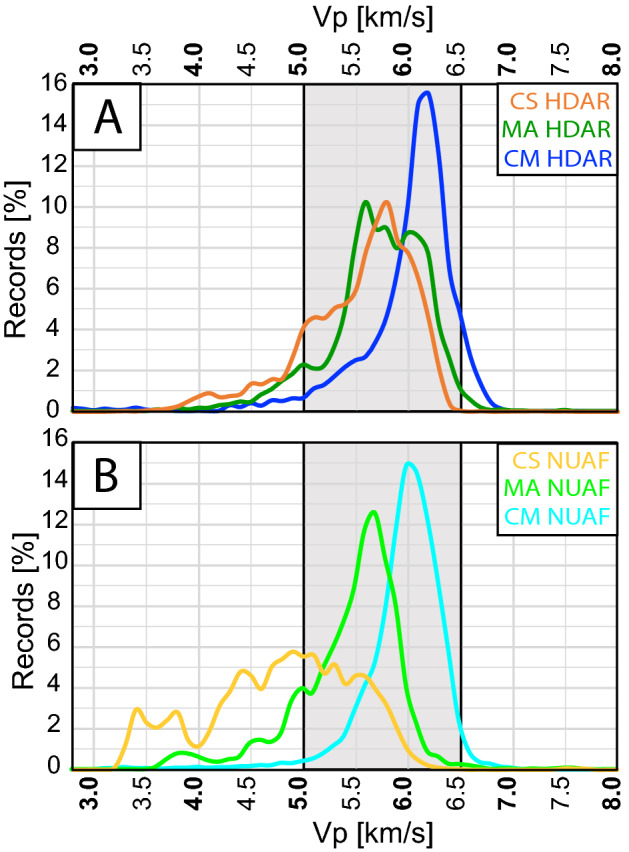
Comparison of frequency histograms normalized respect to the total number of measurements (see text for a detailed explanations) for the three carbonates litho-stratigraphic units. (**A**) Normalized frequency histograms of Calcare Massiccio (Blue), Maiolica (Green) and Calcareous Scaglia (Orange) measured in the Highly-Deformed Apennine Ridge (HDAR). (**B**) Normalized frequency histograms of Calcare Massiccio (Cyan), Maiolica (light Green) and Calcareous Scaglia (Yellow) measured in the nearly-Undeformed Adriatic Foreland (NUAF). For clarity, we normalized the frequency values so that the sum of the reported frequencies for each lithostratigraphic unit pertaining to each of the structural domain, is equal to 100. In other words, we plot the data so that a value of 10 in the y axis and 6.0 km/s in the x axis in the curves in Fig. 8 means that the 10% of the measurements recorded 6.0 km/s.

The frequency analysis of SLV shows that seismic velocities of the three investigated lithologies are mainly in the range 5.0–6.5 km/s for both domains (Fig. 8A,B). MA and more clearly CS formations, show a wider distribution of velocities in NUAF than in HDAR due to the larger records of Vp < 5.0 km/s (Fig. 8B). Since no compositional or stratigraphical variations have been ever reported between the two structural domains for MA and CS^35,67,77^, the wider velocity range in NUAF may be related to the effect of the tectonic environment on diagenesis. Previous works^78,79^ suggest that pressure accelerate diagenesis by enhancing compaction. Larger stresses recorded in HDAR than those in NUAF^18^ may produce a more efficient diagenesis resulting in a more homogeneous velocity distribution. On the contrary, slower diagenesis may have affected the pelagic limestones in NUAF due to the lower stress^18^, explaining the higher number of small recorded velocities with Vp < 5.0 km/s (Fig. 8B). Interestingly, this effect becomes almost absent in CM, possibly due to the fact that CM was deposited in a shallow-water marine environment. This environment favors early diagenesis by cementation and lithification in the near surface^78,80^, before burial and the onset of tectonic stresses.

## Conclusions

Based on our results and interpretations on seismic velocity variations at crustal scale, we can conclude that:

- Interval velocity derived from check shots (IV) and sonic logs velocities (SLV) give comparable results for the tight carbonate rocks investigated in this paper despite the different volume of rock sampled (decimeter-scale for SLV, up to hundreds of meter-scale for IV).
- The presence of lithological (e.g. bedding) and structural (e.g. fractures and stylolites) anisotropies controls the velocity dependence with depth. Increasing lithostatic load (e.g. depth) causes a reduction in structural porosity and the progressive increase of velocity in particular in the thin-layered limestone (CS), while velocities in the massive limestones CM are not affected by depth.
- Despite local variations, higher velocities are measured in the mountain range (HDAR) with respect to the foreland (NUAF) suggesting that the tectonic stress plays a key role in controlling Vp of layered carbonates. In particular, tectonic stress may enhance diagenetic processes and favor the development of lithological and structural anisotropy.

The observed velocity variations from the low deformed foreland domain (NUAF) to the highly deformed mountain range domain (HDAR) reach ~ 10% for the marly thin-layered limestones (CS). This variation corresponds to an increase in the Young’s Modulus (E) of ~ 20% suggesting that HDAR carbonates are stiffer than NUAF. Moreover, the counterintuitive, unexpected result of the increase of velocity in the high deformation domain, usually intensely fractured, suggests that fracturing alone plays a minor role in decreasing velocities of carbonates at depth. These results have a strong impact on the development of velocity and mechanical models for seismically active areas where earthquakes nucleate and propagate through carbonate multilayers, and for the analysis of deep and fractured carbonate reservoirs.

## Supplementary Information

Supplementary Materials.

Supplementary Table 1.

Supplementary Table 2.

Supplementary Table 3.

Supplementary Legends.
