# Supplementary material for: Lithological and stress anisotropy control large-scale seismic velocity variations in tight carbonates
Source: Sci Rep. 2021 May 4;11:9472. doi: 10.1038/s41598-021-89019-4 (PMC8096945; doi:10.1038/s41598-021-89019-4)
Supplement: Supplementary file 1 — Supplementary Materials. [file 41598_2021_89019_MOESM1_ESM.docx]

Supplementary Material – Statistical analysis

The exponential distribution of Vp as a function of depth [y=b1 exp(b2)+b3, where y is Vp and x is the depth] proposed by Athy in 1930 (Ref 54) fits satisfactory well our data, in agreement with previous literature and theory (REF 8,23,47,54,65). We used an extensive new dataset to obtain average trends of Vp values with depth and then, by considering the best-fit of each group of data (CM, MA, CS) with this exponential function (Table S3), we highlighted and discussed the outlier values. Here we report the details of the conducted statistical analysis reported in Figure 6 and Table S3.

The best-fit reported in Figure 6 and Table S3 can be affected by 3 main factors:

1. the uncertainties of the measurements
2. the choice of a single value (either SLV, IV or the average) for boreholes having both measurements (IV and SLV)
3. the weights to be applied in the best-fit procedure to account for the uncertainties

**1. Uncertainties:**

In general, there are different source of uncertainties affecting the data that can be included. In our analysis, uncertainties estimations come from different instruments and procedures used to retrieve the Vp values (punctual measurements with “Sonic log” (SLV) versus average measurements with “Check-shot” called interval velocities (IV) in this work). In particular, the variability of punctual Vp measurements depends also on the thickness of each lithology. In this work, we decided to use the most conservative choice to represent what we call uncertainties. For the SLV we computed the standard deviation of the Vp variation for each specific lithology shown in Figures 3A, 4A, 5A. In this case the SLV instrument error is hidden in this variability (Ref. 47). For the Check-shots (IV) we consider as uncertainties the error coming from the measurements and instrument procedure. In the literature (Ref. 47) it is reported that the error associated to this methodology, in general, can range between 1- 2% of the absolute values. We thus assumed an error of the 2% for IV. Figure S1 (and Figure 6) shows the estimates and their associated uncertainties.


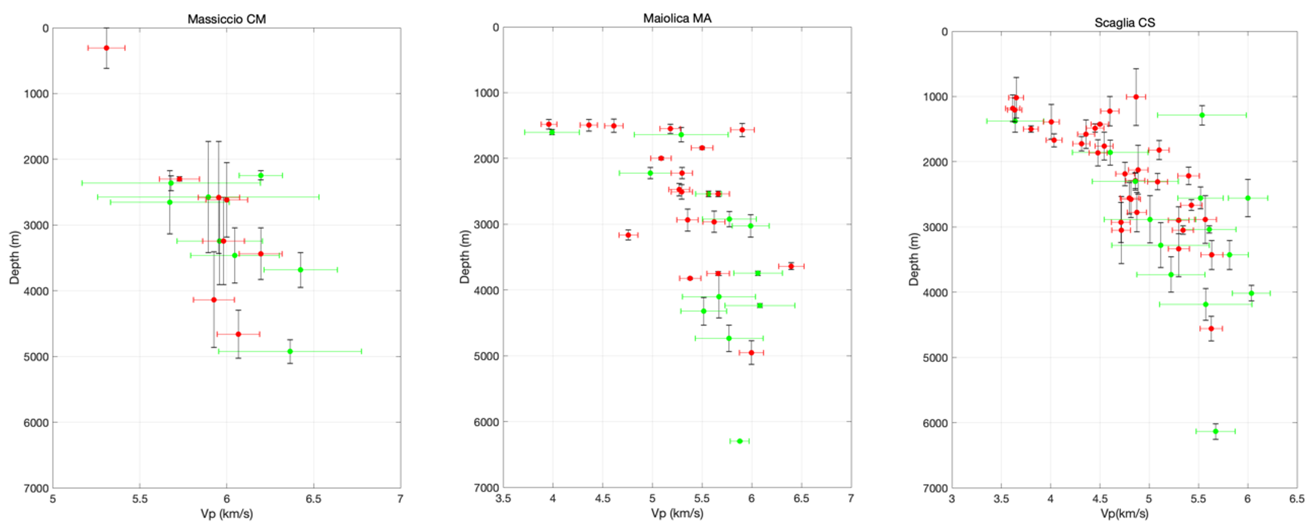


**Figure S1:** IV (red symbols) and SLV (green symbols) measurements for the three group of data. The black vertical bars represent the thickness of each lithology, Horizontal red and green bars represent respectively the IV data errors (2% following47) and the standard deviations of the SLV data.

**2. Redundant measurements:**

For 6 wells (see Figure 1) both SLV and IV were available. Keeping both measurements in the statistical analysis would have obviously biased the results since the same borehole data would have been counted twice. Thus, for the wells having both the measurements, we tested the procedure to retrieve the best-fit with the exponential function proposed by Athy 1930 considering only one dataset (either SLV or IV) or considering an average measurement of the two (in this case errors are the sum of the single errors, due to the propagation error theory). Figure S2 shows that the variation of the best-fit changing the assumption for the wells having both the estimates is very small (green, red and blue curves as explained in the legend of Fiigure S2)


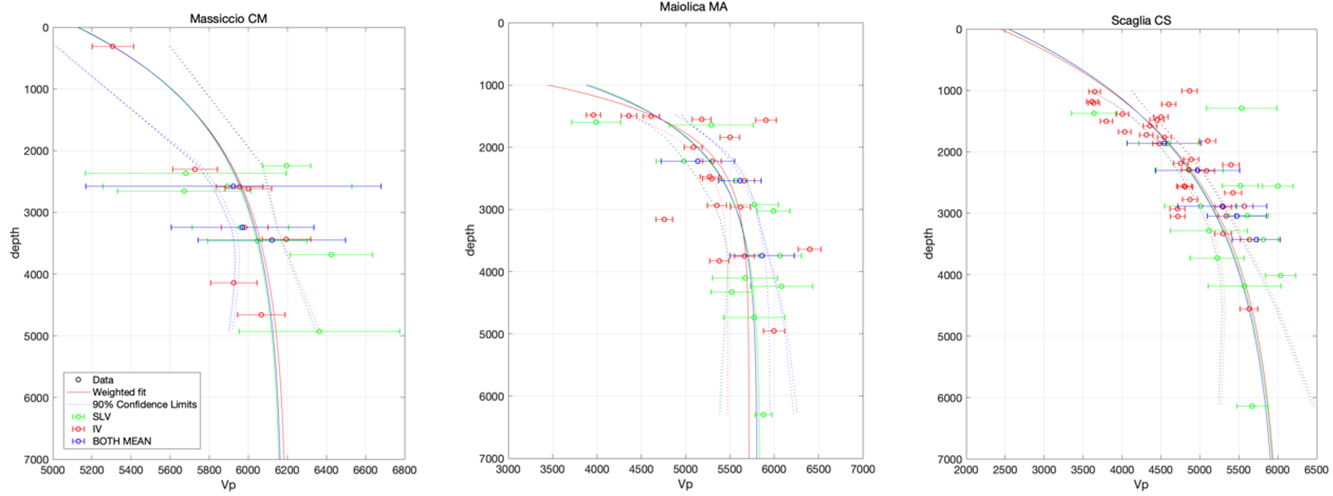


**Figure S2:** Green symbols are for Sonic logs data (SLV), Red symbols are for Check shot (IV) while Blue symbols are the average of the two when both measurements were available. Horizontal red and green bars represent respectively the IV data errors (2% following47) and the standard deviations of the SLV data while blue horizontal bars represent the sum of the errors, due to the propagation error theory. To infer the best fit with an exponential function, the objective function implemented in Matlab has been applied to the available data. Green, Red and Blue bars represent the best fits by using respectively only SLV, only IV or the average values for boreholes having both measurements. Colored dashed lines represent the 90% confidence limit derived from each analysis.

Since different choices would not significantly change the results (Figure S2), for boreholes having both measurements, we use the SLV data due the larger number of measurements respect to check shot. Moreover, the use of the SLV allows us to include a larger uncertainty to these values.

**3. Weights:**

The effect of uncertainties in our analysis can be appreciated comparing the best-fit with and without weights. We underline that the weights included in the inversion procedure correspond to the inverse of the measurement uncertainties shown in Figure 6.


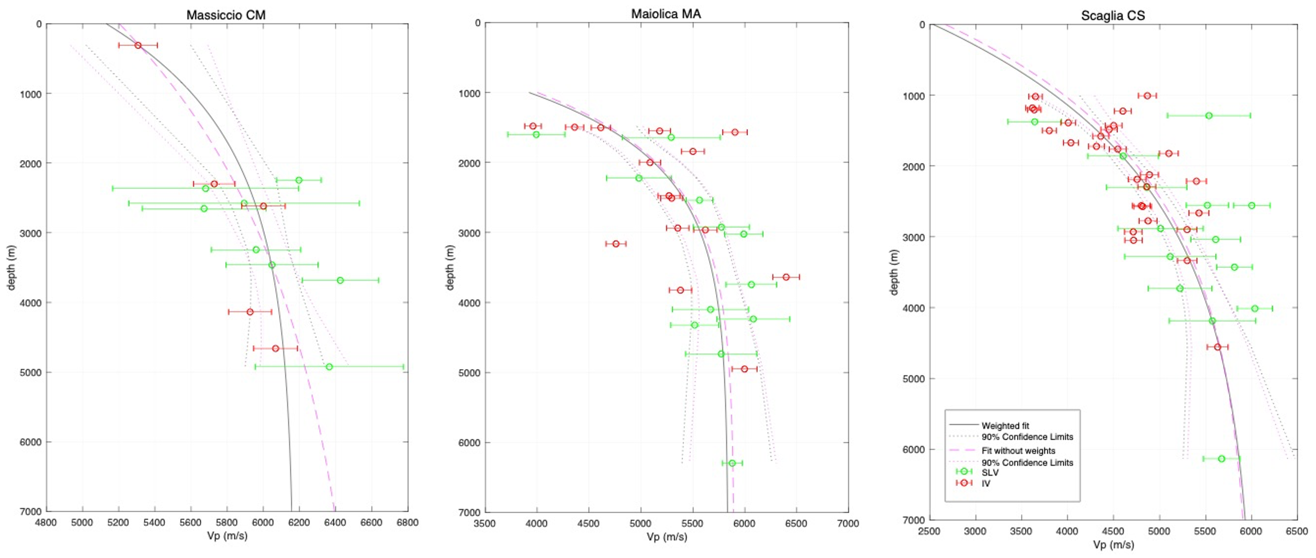


**Figure S3:** Mean velocities vs mean depths for boreholes that drilled Calcare Massiccio, Maiolica and Calcareous Scaglia. Horizontal red and green bars represent respectively the IV data errors (2% following47) and the standard deviations of the SLV data. Purple dashed line represents unweighted fits whilst black solid lines represent weighted fits, where weights correspond to the inverse of the measurement uncertainties. Purple and black dotted lines represent the 90% confidence limit derived from the two analyses, respectively unweighted and weighted.

We thus included the weights in the best-fit procedure to account for the uncertainties. The larger differences among the two best-fits come from the CM dataset (Figure S3) where few data were available, in particular at depth. However, those difference would slightly affect the Vp differences showed in Figure 7 and thus the main conclusions of the paper.
