# Supplementary material for: Lithological and stress anisotropy control large-scale seismic velocity variations in tight carbonates
Source: Sci Rep. 2021 May 4;11:9472. doi: 10.1038/s41598-021-89019-4 (PMC8096945; doi:10.1038/s41598-021-89019-4)
Supplement: Supplementary file 5 — Supplementary Legends. [file 41598_2021_89019_MOESM5_ESM.docx]

Supplementary information

**Lithological and stress anisotropy control large-scale seismic velocity variations in tight carbonates.**

**F. Trippetta^1^, M.R. Barchi^2^, E. Tinti^1,3^, G. Volpe^1^, G. Rosset^4^ and N. De Paola^5^**

^1^Sapienza Università di Roma, Dipartimento di Scienze della Terra (DST), Rome, 00185, Italy

^2^Università di Perugia, Dipartimento di Fisica e Geologia, Perugia, 06123, Italy

^3^Istituto Nazionale di Geofisica e Vulcanologia, 00143 Rome, Italy

^4^Università di Trieste, Dipartimento di Matematica e Geoscienze, Trieste, 34128, Italy

^5^Durham University, Department of Earth Sciences, Durham, DH13LE, United Kingdom

Corresponding author: Fabio Trippetta ([fabio.trippetta@uniroma1.it](mailto:fabio.trippetta@uniroma1.it))

**Supplementary Material:** Methodology details of the performed statistical analysis

**Supplementary Table S1:** Velocity data used for this work

**Supplementary Table S2:** Averaged and weighted velocities for all boreholes. Green is for SLV data and red is for IV. *Tavullia 1 drilled the CM twice: from 3044 m to 3.830 m (average depth 3450 m) and from 4298 m to 5025 m (average depth 4662 m) where only IV data are available for the shallower interval and both IV and SLV are available for the deepest interval. The mean value for each formation has been calculated by averaging the mean velocity of each well, being weighted with respect to the drilled thickness. For the areal mean calculations (HDAR and NUAF means), when both IV and SLV data were present we used only SLV data to avoid redundancy. ** This datum has been excluded from calculations since clearly related to acquisition problems.

**Supplementary Table S3:** Best fit Velocity/depth equations parameters calculated for each group (CM, MA and CS)
